# Large magnetocapacitance beyond 420% in epitaxial magnetic tunnel junctions with an MgAl_2_O_4_ barrier

**DOI:** 10.1038/s41598-022-11545-6

**Published:** 2022-05-16

**Authors:** Kenta Sato, Hiroaki Sukegawa, Kentaro Ogata, Gang Xiao, Hideo Kaiju

**Affiliations:** 1 Faculty of Science and Technology, Keio University, Yokohama, Kanagawa 223-8522 Japan; 2 Research Center for Magnetic and Spintronic Materials, National Institute for Materials Science, Tsukuba, Ibaraki 305-0047 Japan; 3 Department of Physics, Brown University, Providence, RI 02912 USA; 4 Center for Spintronics Research Network, Keio University, Yokohama, Kanagawa 223-8522 Japan

**Keywords:** Materials science, Condensed-matter physics, Spintronics, Physics, Electronics, photonics and device physics, Electronic and spintronic devices

## Abstract

Magnetocapacitance (MC) effect has been observed in systems where both symmetries of time-reversal and space-inversion are broken, for examples, in multiferroic materials and spintronic devices. The effect has received increasing attention due to its interesting physics and the prospect of applications. Recently, a large tunnel magnetocapacitance (TMC) of 332% at room temperature was reported using MgO-based (001)-textured magnetic tunnel junctions (MTJs). Here, we report further enhancement in TMC beyond 420% at room temperature using epitaxial MTJs with an MgAl_2_O_4_(001) barrier with a cation-disordered spinel structure. This large TMC is partially caused by the high effective tunneling spin polarization, resulted from the excellent lattice matching between the Fe electrodes and the MgAl_2_O_4_ barrier. The epitaxial nature of this MTJ system sports an enhanced spin-dependent coherent tunneling effect. Among other factors leading to the large TMC are the appearance of the spin capacitance, the large barrier height, and the suppression of spin flipping through the MgAl_2_O_4_ barrier. We explain the observed TMC by the Debye-Fröhlich modelled calculation incorporating Zhang-sigmoid formula, parabolic barrier approximation, and spin-dependent drift diffusion model. Furthermore, we predict a 1000% TMC in MTJs with a spin polarization of 0.8. These experimental and theoretical findings provide a deeper understanding on the intrinsic mechanism of the TMC effect. New applications based on large TMC may become possible in spintronics, such as multi-value memories, spin logic devices, magnetic sensors, and neuromorphic computing.

## Introduction

Magnetocapacitance (MC) effect has been getting much attention due to the fascinating spin physics and potential applications^1–4^. MC effect appears in a system with broken time-reversal and space-inversion symmetry, such as in multiferroic materials^5,6^ and spintronic devices^2–4,7–10^. More recently, it has been observed in magnetic supercapacitors^11,12^, organic heterojunctions^13,14^, graphene-based two-dimensional (2D) materials^15,16^, and three-dimensional (3D) topological insulators^17,18^. These materials or devices have prospects for applications in high-performance magnetic sensors, memory devices, and data storage systems^2–9,11–14^.

Magnetic tunnel junctions (MTJs)^4,8,19^ are a common spintronic device and can exhibit a large MC effect at room temperature, often referred to as tunnel magnetocapacitance (TMC)^2–4,7–9^. MTJs are also known to show a large tunnel magnetoresistance (TMR) effect^20–26^. TMC’s unique properties include a relative larger TMC (measured in percentage) over TMR at some specific frequencies^4^, good thermal stability in TMC^19^, and robustness of TMC against a bias voltage as opposed to a bias-induced TMR drop^8^. Recently, by optimizing appropriate frequencies and bias voltages, a large TMC of 332% was achieved in MgO-based MTJs at room temperature^27^. The core structure of MTJs is Co_40_Fe_40_B_20_/MgO/Co_40_Fe_40_B_20_, leading to a *textured* CoFe/MgO/CoFe(001) interfacial structure promoted by an annealing process. Based on the Debye-Frölich (DF) modelled calculation, the spin polarization which contributes to TMC is 47.7% in this MTJ system.

The lattice-mismatch between rock-salt MgO and bcc ferromagnetic (FM) electrodes (Fe, CoFe, etc.) prevents further improvement in the properties of MgO-based MTJs. An alternative barrier, a spinel MgAl_2_O_4_ (MAO), is a good candidate for MTJs, as the new barrier maintains the Δ_1_-preferential coherent tunneling effect, which is necessary acquiring a large TMC effect^28–35^. The crystalline MAO barrier is superior to MgO in in-plane lattice matching with bcc-electrodes—~ 0.404 nm in MgAl_2_O_4_(200) versus ~ 0.421 nm in MgO(100)^29,30^. However, the first-principle band calculation predicts that an effective dc-tunneling spin polarization through Fe/MgAl_2_O_4_/Fe(001) is much smaller than that through Fe/MgO/Fe(001) due to the appearance of additional Δ_1_ conduction channels in minority spin states originating from a band-folding effect of Fe^36^. Introducing cation site disordering into the spinel structure that accompanies the crystal symmetry change from $Fd\overline{3} m$ to $Fm\overline{3} m$ or $F\overline{4} 3m$ (cation-disordered spinel structure) reduces the lattice unit size to half of the primitive MgAl_2_O_4_, which effectively suppresses the band-folding effect^30,37^; a TMR ratio of Fe/*cation-disordered* MAO/Fe(001) reaches ~ 436% at low temperature (245% at room temperature)^32^, which is far beyond the calculated upper limit of ~ 160% in Fe/*cation-ordered* MAO/Fe(001). Since the corresponding dc spin polarization is over ~ 60%, a giant TMC beyond conventional values could be observed.

In this work, we report the largest TMC of 426% in MTJs with cation-disordered spinel MAO barriers at room temperature. The frequency characteristics and voltage dependence of TMC can be well explained by the calculations based on modified DF model combined with Zhang formula, sigmoid function, parabolic barrier approximation (PBA), and spin-dependent drift–diffusion (SDD) mechanism. This means that the large TMC is attributed to the spin capacitance appeared in Fe/MAO interfaces, the formation of MAO high barrier and the suppression of spin flipping through an MAO barrier. The calculation also predicts that TMC could reach 1000% in MTJs with a high spin polarization of 80%. Our findings open a new avenue to the development of spintronics applications.

## Results and discussion

### Device structures

**Figure 1:**
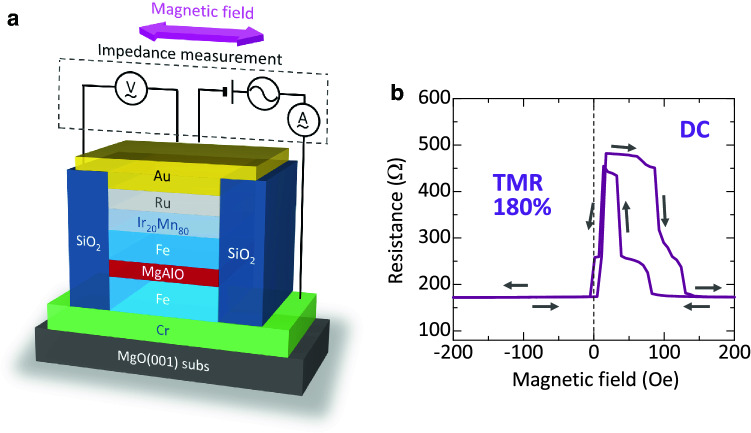
Experimental set-up and device structure. (**a**) Schematic of a patterned MAO-based MTJ with the stack structure. The measurement set-up for TMC is also shown. The magnetic field is applied along the Fe[100]||MAO[110] direction. (**b**) TMR curve measured by a dc four probe method.

Figure 1a shows the device structure prepared by a high vacuum dc/rf magnetron sputtering system, in a base pressure of 5 × 10^−7^ Pa, with the following layer sequence: MgO(001) single-crystal substrate/Cr (40)/Fe (30)/Mg (0.45)/Mg_19_Al_81_ (*d*_MgAl_ ~ 1.22–1.25)/ oxidation/Fe (7)/Ir_20_Mn_80_ (IrMn) (12)/Ru (12) (unit: nm). Details of the device fabrication procedure are described in the Experimental Section. After the growth of the multilayer stack, the MTJ structures were patterned into a junction area of 5 × 10 μm^2^ with an elliptical shape using typical microfabrication processes including photolithography and Ar ion-beam etching.

The current–voltage (*I-V*) characteristics and TMR curves in MTJs were measured using a dc four-probe method at room temperature. The magnetic field was applied along Fe[100] || MAO[110] up to 300 Oe. A typical TMR curve is shown in Fig. 1b. A large TMR ratio of 180% is observed, indicating the achievement of optimum oxidation of the MAO interfaces. Here, the TMR ratio is defined by $(R_{{\rm{AP}}} - R_{\rm{P}} )/R_{\rm{P}}$, where *R*_P(AP)_ is the resistance in the parallel (anti-parallel) magnetization states of both FM layers. The corresponding dc tunneling spin polarization is 68.8%. The large TMR also indicates the formation of an epitaxial MAO barrier^30^. In this study, two MTJs with a TMR ratio of 174% (sample A, *d*_MgAl_ = 1.25 nm) and 183% (sample B, *d*_MgAl_ = 1.22 nm) were used for TMC measurements.

### Frequency characteristics of TMC under no bias voltage

**Figure 2:**
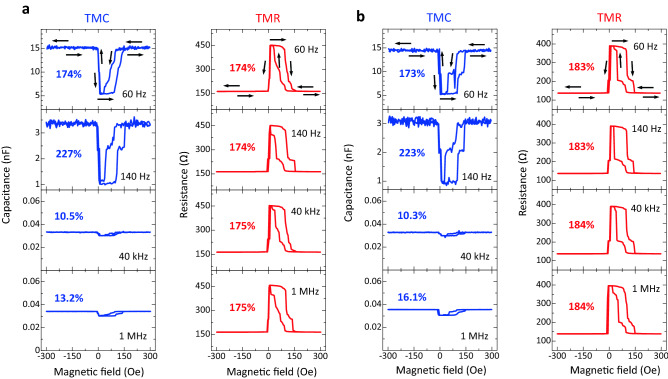
TMC and TMR curves under no bias voltage. (**a**) TMC and TMR curves at 60 Hz, 140 Hz, 40 kHz, and 1 MHz for an Fe/MAO/Fe MTJ (sample A) (Bias voltage ~ 0 V). At 140 Hz, the TMC ratio is 227%, greater than the value in MgO-based MTJs (172%). (**b**) TMC and TMR curves for sample B.

**Figure 3:**
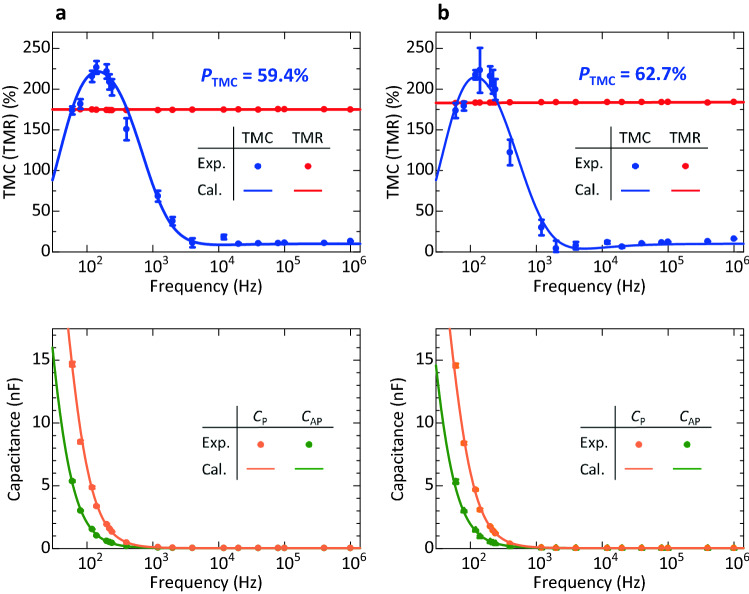
Frequency characteristics of TMC, TMR, and *C*_P(AP)_. (**a**,**b**) TMC, TMR (upper panels), and *C*_P(AP)_ (lower panels) for an Fe/MAO/Fe MTJ of samples A and B, respectively. The solid points are experimental data and solid lines represent the calculation results based on DF model, Eq. (1). The error bars are standard deviations. The experimental data are reasonably fitted by the model for both samples.

Figure 2 shows the TMC and TMR curves at 60 Hz, 140 Hz, 40 kHz, and 1 MHz for samples A and B, respectively. The bias voltage is ~ 0 V. Positive TMC and TMR effects are observed at each frequency. TMR ratios are independent of the frequency, and are 174% and 183% for samples A and B, respectively. Here, the TMC ratio is defined by $(C_{\rm{P}} - C_{{\rm{AP}}} )/C_{{\rm{AP}}}$, where *C*_P(AP)_ is the capacitance in the parallel (anti-parallel) magnetization states for both FM layers. The TMC ratio increases with increasing the frequency in the low frequency region, and it reaches 227% (223%) at 140 Hz for sample A (B). The TMC of 227% (223%) is larger than the conventional value of 172%, which is observed in MgO-based MTJs^27^. As increasing the frequency, the TMC decreases to 10.5% (10.3%) at 40 kHz, and it slightly increases to 13.2% (16.1%) for sample A (B). Here we note that the TMC and TMR curves are slightly different with different frequency. Although the reason is not clear at the present stage, the TMC and TMR are sensitive to the magnetization states of Fe layers in Fe/MAO interfaces. The magnetization states of Fe layers slightly change depending on the interfacial structures, magnetic pinning, domain wall motion, etc. These factors could cause the slight changes in TMC and TMR curves. As is well known, the spin transfer torque (STT) switching is observed in MTJs with a junction area of smaller than a few 100 nm scale (single domain scale for in-plane magnetized films) and with a thin free layer (less than a few nm). For examples, it is observed in CoFe/MgO/CoFe MTJs with a junction area of 100 × 150 nm^2^, 100 × 200 nm^2^, or 100 × 300 nm^2^ and with a free-layer thickness of 1.8–2.6 nm^38^, and in CoFe/MgO/CoFe MTJ pillars with a diameter of 40 or 100 nm and with a free-layer thickness of 1.0 − 1.7 nm^39^. In contrast, the junction area of the MTJs fabricated in this study is as large as 5 × 10 μm^2^ and the thickness of the Fe free layer is 30 nm. Therefore, it is considered that STT switching cannot be expected in our MTJs. The voltage-controlled magnetic anisotropy (VCMA) can be observed in MTJs with an FM layer thickness of less than a few nm. For examples, it is demonstrated in Fe/MgO structures with 2 − 4 Fe monolayers with strong perpendicular magnetic anisotropy^40^, and in Fe(0.5)/Ir(0.1)/CoFe(0.1)/MgO (numbers are nominal thicknesses in nanometers) MTJs^41^. Therefore, it is considered that VCMA cannot be significant in our MTJs that have thicker Fe layers and in-plane anisotropy. The frequency characteristics of TMC, TMR, and *C*_P(AP)_ are shown in Fig. 3. *C*_P(AP)_ at frequency *f* is calculated by the following DF model:

1 $$C_{{\rm{P(AP)}}} = {\text{Re}} \left[ {C_{{\infty ,\,\rm{P(AP)}}} + \frac{{C_{{0,\,\rm{P(AP)}}} - C_{{\infty ,\,\rm{P(AP)}}} }}{{1 + (i2\pi {\kern 1pt} f\tau_{{\rm{P(AP)}}} )^{{\beta_{{\rm{P(AP})}} }} }}} \right],$$

where $C_{{\infty ,\rm{P(AP)}}}$ and $C_{{0,\rm{P(AP)}}}$ are the high-frequency and DC capacitances, *τ*_P(AP)_ is the relaxation time, and *β*_P(AP)_ is the exponent showing the distribution of relaxation time, respectively, for the P (AP) configuration. The relation between *τ*_P_ and *τ*_AP_ is given by

2 $$\tau_{{\rm{AP}}} = \frac{{1 + P_{{\rm{TMC}}}^{2} }}{{1 - P_{{\rm{TMC}}}^{2} }}\tau_{\rm{P}} ,$$

where *P*_TMC_ is the spin polarization, contributing to TMC, inside the FM layer^4,42^. TMR is calculated using the Julliere formula^43^. As shown in Fig. 3, the calculation results of TMC, *C*_P(AP)_, and TMR fit to the experimental data well. The parameters obtained in the fitting calculation are shown in Table S1. We obtain *P*_TMC_ of 0.594 (0.627) and *P*_TMR_, contributing to TMR, of 0.682 (0.691) for sample A (B) by the fit. Here we note that *P*_TMC_ is lower than *P*_TMR_ for samples A and B. The difference between *P*_TMC_ and *P*_TMR_ is attributed to the different penetration lengths of spin-dependent carriers (electrons or holes inside Fe) for TMC and TMR. The penetration length of TMC is considered to be longer than that of TMR^4^. The spin polarization of the interfacial FM atoms between FM/insulator layers is higher than that of inner atoms from the interface atoms due to the two-dimensional effect^44–47^. In our cases, the spin polarization of the interfacial atoms in Fe layers is also considered to be higher than that of inner atoms from the Fe/MAO interface. Therefore, *P*_TMC(TMR)_ is low (high) for a long (short) penetration length. Our calculation also indicates that higher *P*_TMR_ generally gives higher *P*_TMC_. Since the spin polarizations *P*_TMC_ and *P*_TMR_ of sample B are larger than that of sample A, the bias voltage dependences of TMC and TMR in sample B are investigated (as for sample A, see the supplementary information).

### Observation of a large TMC by biasing dc voltage

**Figure 4:**
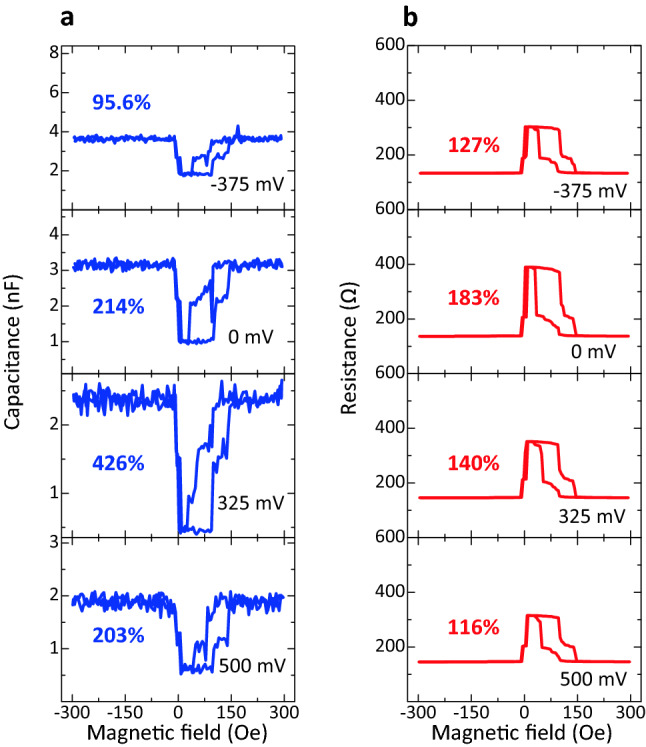
Voltage dependence of TMC and TMR curves. (**a**) TMC curves and (**b**) TMR curves of an Fe/MAO/Fe MTJ (sample B).

**Figure 5:**
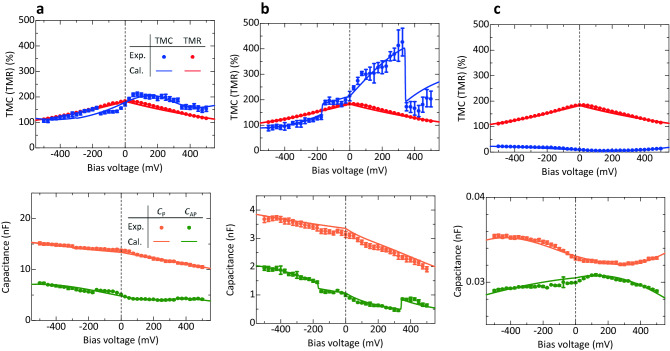
Voltage dependence of TMC, TMR, and *C*_P(AP)_ for an Fe/MAO/Fe MTJ (sample B). (**a**) At 60 Hz, (**b**) at 140 Hz, and (**c**) at 40 kHz. The solid circles are experimental data and the solid lines are the calculation results. The calculation is performed using SDD model and DF model combined with PBA and Zhang-sigmoid formula (see text).

Figure 4 shows the bias voltage dependence of TMC and TMR curves at 140 Hz in sample B. TMR decreases from 183% to 116% with increasing the voltages up to 500 mV in the positive bias region. The same tendency can be seen in the negative region. The reduction of TMR can be attributed to spin flipping process in the AP configuration by interface magnetic excitation^48^ in addition to the effect of the Fe band structure^49^. In contrast, the TMC ratio increases with increasing the voltage in the positive bias region, and it reaches 426% at 325 mV. A TMC of 426% is the largest value ever reported for MTJs. As increasing the voltage higher than 325 mV, the TMC ratio decreases to 203% at 500 mV. In the negative bias region, the TMC ratio decreases with the increase of bias voltage. Figure 5 shows the bias dependence of TMC, TMR, and *C*_P(AP)_ at 60 Hz, 140 Hz, and 40 kHz. The TMC and TMR curves at 60 Hz and 40 kHz are shown in Fig. S1. As described above, at 140 Hz, the TMC reaches 426% at 325 mV in the positive bias region, and then it rapidly drops at a higher voltage of 325 mV. In the negative bias region, the TMC shows plateau behavior from 0 to − 150 mV, and then it drops sharply. After that, it slightly decreases. At 60 Hz, in contrast, the TMC shows the almost the same behavior as the TMR. At 40 kHz, the TMC tends to increase with increasing the voltage in the bipolar bias regions. The model calculation of the bias dependence of the TMC and *C*_P(AP)_ is performed using SDD model and DF model combined with PBA and Zhang-sigmoid formula. In the modified DF model, the capacitance based on PBA and Zhang-sigmoid formula for the P (AP) configuration under the bias voltage *V* can be expressed by

3 $$C_{{\rm{P(AP)}}}^{{\rm{DF} - \rm{ZSP}}} (f,V) = \frac{1}{{1 - \frac{e(1 - \kappa )V}{{4\phi_{{0,\rm{P(AP)}}} }}}}{\text{Re}} \left[ {C_{{\infty ,\,\rm{P(AP)}}} + \frac{{C_{{0,\,\rm{P(AP)}}} - C_{{\infty ,\,\rm{P(AP)}}} }}{{1 + (i2\pi {\kern 1pt} f\tau_{{\rm{P(AP),}V}} )^{{\beta_{{\rm{P(AP})}} }} }}} \right].$$

Here, the coefficient of the real part of the capacitance in Eq. (3) is determined by PBA, which describes the bias voltage dependence of the effective barrier thickness^50^. *ϕ*_0,P(AP)_ is the barrier height in the absence of the bias voltage for the P (AP) configuration, $e$ is the electron charge, and *κ* is the parameter determined by the contributing ratio of the dynamic capacitance to the overall capacitance. $C_{{\infty ,\rm{P(AP)}}}$, $C_{{0,\rm{P(AP)}}}$, and *β*_P(AP)_ in the real part of the capacitance are the same parameters in Eq. (1). The relaxation time can be expressed by

4 $$\tau_{{\rm{P(AP)},\;V}} = \frac{1}{{1 + K_{{\rm{P(AP)}}} (1 - \kappa )Vg_{{\rm{P(AP)}}} (V)}}\tau_{{\rm{P(AP)},\;0}} .$$

Here *K*_P(AP)_, which is appeared in Zhang model, is the parameter determined by Curie temperatures of FM layers, the density of states of itinerant electrons in FM layers, and direct and spin-dependent transfers and spin quantum number within the framework of the transfer Hamiltonian in the system of FM/insulator/FM. *τ*_P(AP),0_ is the relaxation time for the P (AP) configuration under no bias voltage. The sigmoid function under the application of bias voltage can be expressed by

5 $$g_{{\rm{P(AP)}}} (V) = \frac{1}{{1 + \exp \left[ { - \alpha_{{\rm{P(AP)}}} \left\{ {\left. {(1 - \kappa )V - V_{{0,\rm{P(AP})}} } \right\}} \right.} \right]}},$$

where *α*_P(AP)_ is the broadening of the contributing rate of dynamic capacitance to the overall capacitance in the P (AP) configuration and *V*_0,P(AP)_ is the voltage for spin flipping. Thus, the Zhang-sigmoid formula gives the bias voltage dependence of the relaxation time^27^. Based on the SDD model for the static case, the spin capacitance can be given by

6 $$C_{{\rm{P(AP), }V}}^{{\rm{SDD}}} = eS\frac{{n_{{0,\,\rm{P(AP)}}} \lambda }}{\kappa \left| V \right|},$$

where *S* is a junction area, *λ* is a characteristic screening length and *en*_0,P(AP)_ is a screening charge density at the interface. Therefore, the total capacitance $C_{{\rm{P(AP),}V}} (f)$ at the bias voltage *V* is given by

7 $$C_{{\rm{P(AP), }V}} (f) = \left( {\frac{1}{{C_{{\rm{P(AP), }V}}^{{\rm{DF - ZSP}}} (f)}} + \frac{1}{{C_{{\rm{P(AP), }V}}^{{\rm{SDD}}} }}} \right)^{ - 1} .$$

**Figure 6:**
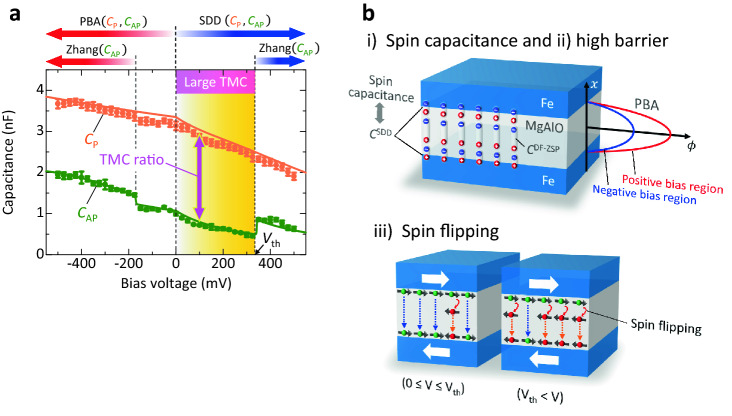
Mechanism of a large TMC. (**a**) Capacitance *C*_P(AP)_ in the P (AP) configuration for an Fe/MAO/Fe MTJ (sample B) at 140 Hz. The large TMC originates from the appearance of the spin capacitance which is described by SDD model. (**b**) Schematic of the mechanism of large TMC: (i) the spin capacitance appears in the Fe/MAO interface, (ii) the barrier height of MAO is high at 1.8–2 eV, and (iii) the spin flipping does not occur in the bias region from 0 to 325 mV.

The bias voltage dependence of the TMC ratio at frequency *f* can be calculated using Eqs. (3)−(7). As can be seen in Fig. 5, the experimental data provide an excellent fit to the calculation results in the entire bias region at each frequency. The parameters obtained in the fitting calculation are shown in Table S2. Here we discuss the mechanism of a large TMC based on the fitting analysis. As shown in Fig. 5b, since both the *C*_P_ and *C*_AP_ decrease with increasing the voltage from 0 to 325 mV, the large TMC is observed in this region. Based on the PBA, a biasing voltage reduces the effective barrier thickness of the insulating layer, resulting in the increase of *C*_P(AP)_. Also, based on the Zhang-sigmoid model, the relaxation time becomes shorter with increasing the voltage, and it rapidly shortens near the spin-flipping voltage *V*_0,P(AP)_. This results in the gradual increase of *C*_P(AP)_ including the rapid increase at the threshold voltage *V*_0,P(AP)_. Since the observed *C*_P_ and *C*_AP_ decrease with increasing the voltage, the PBA and Zhang-sigmoid model do not mainly contribute to the capacitance. In contrast, the SDD model describes the spin capacitance that appears in the FM/insulator interface due to the deference in chemical potential between both the FM layers. Since the spin capacitance can be expressed by Eq. (7), the *C*_P(AP)_ decreases with increasing the voltage. The decrease of capacitance by biasing agrees with the observed results. This means that the SDD model is dominant in the bias region from 0 to 325 mV, indicating that the large TMC originates from the appearance of spin capacitance (See Fig. 6).

We also note that the asymmetry behavior can be seen in the bias dependences of TMC and *C*_P(AP)_. The *C*_P_ and *C*_AP_ increase with increasing the voltage in the negative bias region. This means that the PBA mainly contributes to the observed capacitance (See Fig. 6a). This is consistent with the fitting analysis that the barrier height *ϕ*_0,P(AP)_ in the negative bias region is lower than that in the positive bias region as shown in Table S2. As the barrier height is low, the effective barrier thickness becomes thin with applying the voltage. Moreover, we notice that the jump-up of *C*_AP_ can be seen at around + 325 mV and − 175 mV. This indicates that the spin flipping occurs at a higher than + 325 mV and − 175 mV in the positive and negative bias regions, respectively. This is also consistent with the fitting results that the spin-flipping voltage *V*_0,AP_ is + 310 mV and − 153 mV in the positive and negative bias regions, respectively. Therefore, Zhang model contributes to the observed capacitance in addition to the SDD model in the positive bias region and PBA in the negative bias region (See Fig. 6a). Although the method to control the threshold bias *V*_th_ is not clear at the present stage, it could depend on materials; *V*_th_ =  ~ 100 mV for CoFe/MgO/CoFe MTJs^27^ and *V*_th_ =  ~ 300 mV for Fe/MAO/Fe MTJs in this study. The threshold bias could be controlled by changing materials, thicknesses and crystal structures of MTJ layers. As shown in Fig. 5a,c, there is also an excellent agreement between theory and experimental results for the TMC in the entire voltage regions at 60 Hz and 40 kHz. This means that the mechanism of TMC properties can be explained by the fitting analysis in the same manner. As for sample A, the experimental data are also in good agreement with calculation results, as shown in Figs. S2 and S3. The parameters obtained in the fitting calculation are shown in Tables S1 and S2.

### Prediction of an extremely large TMC and high-frequency shift

**Figure 7:**
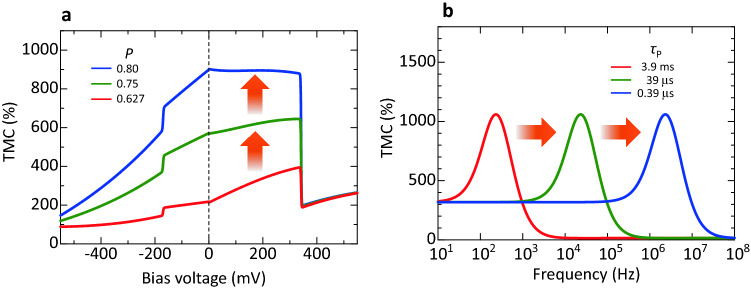
Prediction of an extremely large TMC and high-frequency shift. (**a**) Bias dependence of TMC at 140 Hz with varying spin polarization *P*_TMC_. TMC is enhanced in higher spin polarization. (**b**) Frequency characteristics of TMC at 325 mV with varying the relaxation time *τ*_P_. The large TMC of 1060% is shifted to the high frequency region for short *τ*_P_.

Successful modelling allows us to predict an extremely large TMC by the development of MTJs with a higher spin polarization. Figure 7a shows the calculated voltage dependence of TMC with varying the spin polarization of FM layers. The main parameters used in the calculation are the same as that in the calculation of TMC in sample B at 140 Hz (See Tables S1 and S3, 140 Hz). As can be seen from Fig. 7a, even though the spin polarization *P*_TMC_ only increases from 0.627 to 0.8, the maximum TMC ratio is expected to increase from 393% to 892%. Figure 7b shows the frequency characteristics assuming TMC at *V*_DC_ = 325 mV and *P*_TMC_ = 0.8 with different relaxation times *τ*_P_. The other parameters are the same as in Tables S1 and S3 (140 Hz). The maximum TMC is 1060% at 230 Hz, 23 kHz, and 2.3 MHz for *τ*_P_ of 3.9 ms, 39 μs, and 0.39 μs, respectively. This indicates that the peak of TMC is shifted to a high frequency region on the order of MHz when a short *τ*_P_ in the sub-μs scale is achieved. The DF model suggests that the relaxation time is determined by the oscillation speed of electric dipoles formed near the interfaces between an FM layer and an insulator. The relaxation time is short in a high oscillation speed. For a short relaxation time, the thickness of the insulator should be thinner. Therefore, the formation of a thinner MAO layer is necessary for high-frequency operation. In fact, recently reported papers demonstrate a high-frequency operation of ~ 100 MHz, corresponding to a relaxation time of sub ns, in FeCo-MgF and Co-MgF nanogranular tunneling systems, respectively^9,51^. The high-frequency shift of TMC will be of great importance for future spintronic applications, such as magnetic read heads and memory devices.

In summary, we observed a large TMC of up to 426% in Fe/MAO/Fe MTJs at room temperature, which is the largest value in MTJs. The large TMC originates from a high spin polarization of 62.7% due to the reduced spin scattering process under bias voltage application by excellent lattice matching between Fe and MAO and the appearance of spin capacitance in Fe/MAO interfaces. Additionally, the high barrier height of MAO (1.8–2.0 eV) and the spin flipping suppressed in the low bias region from 0 to 325 mV are important features. These physical pictures can be understood by the static SDD model and dynamic DF model incorporating PBA and Zhang-sigmoid model. This model predicts that the TMC could potentially exceed 1000% in MTJs when a spin polarization reaches 80%. Our theoretical and experimental findings provide new insights into the exact mechanism of the TMC effect in MTJs. The prospect of increasingly larger TMC opens exciting opportunities for new spintronics applications. MTJs endowed with a giant TMR and TMC are superior magnetic and spintronic devices rich in spin-dependent physics and ready for unexplored electrical engineering designs over a wide frequency range.

## Methods

### Preparation of the samples

The MTJs were prepared by using a high vacuum magnetron sputtering system in a base pressure of 5 × 10^−7^ Pa, with the following layer sequence: MgO(001) single-crystal substrate/Cr (40)/Fe (30)/Mg (0.45)/Mg_19_Al_81_ (*d*_MgAl_ = 1.22 and 1.25 nm)/oxidation/Fe (7)/IrMn (12)/Ru (12) (unit: nm). We deposited the Cr, Fe, Mg, IrMn, and Ru layers in DC mode and the MgAl layer in radio frequency (RF) mode with magnetron sputtering under an Ar gas pressure of 0.1 Pa. An MAO barrier was formed by direct inductively coupled plasma (ICP) oxidation of the Mg/MgAl bilayer. The ICP oxidation used an input RF power density of 0.24 W/cm^2^ under an O_2_ + Ar mixture gas (total 6 Pa) for 45 s. The detailed condition is described in Ref.^30^. After the growth of the multilayer stack, the MTJ structures were patterned into a junction area of 5 × 10 μm^2^ with an elliptical shape by using photolithography, Ar ion-beam etching, and SiO_2_ insulation sputtering. The MTJs were annealed at 175 °C in a vacuum furnace (base pressure: ~ 10^−5^ Pa) under a 5 kOe magnetic field for an hour along Fe[100] to induce exchange bias for the top Fe layer.

### Measurements of the voltage-induced TMC

The frequency characteristics and the bias voltage dependence of the TMC and TMR for MTJs were measured by an AC four-probe method using the Agilent Technologies 4284A LCR meter at room temperature. The frequency ranged from 60 Hz to 1 MHz and the bipolar bias voltage was applied up to 500 mV. The AC voltage was set at 50 mV_rms_. The magnetic field was applied along Fe[100] || MAO[110] up to 300 Oe.

## Supplementary Information

Supplementary Information.

## Data Availability

All data generated or analyzed during this study are included in this published article and its supplementary information files.
