# Supplementary material for: Large magnetocapacitance beyond 420% in epitaxial magnetic tunnel junctions with an MgAl2O4 barrier
Source: Sci Rep. 2022 May 16;12:7190. doi: 10.1038/s41598-022-11545-6 (PMC9110733; doi:10.1038/s41598-022-11545-6)
Supplement: Supplementary file 1 — Supplementary Information. [file 41598_2022_11545_MOESM1_ESM.pdf]

Large magnetocapacitance beyond 420% in epitaxial magnetic tunnel junctions with an $MgAl_2O_4$ barrier

---

Supplementary Information

Kenta Sato[1], Hiroaki Sukegawa[2], Kentaro Ogata[1], Gang Xiao[3] and Hideo Kaiju[1,4]

[1]Faculty of Science and Technology, Keio University, Yokohama, Kanagawa 223-8522, Japan

[2]Research Center for Magnetic and Spintronic Materials, National Institute for Materials Science, Tsukuba, Ibaraki 305-0047, Japan

[3]Department of Physics, Brown University, Providence, RI 02912, USA

[4]Center for Spintronics Research Network, Keio University, Yokohama, Kanagawa 223-8522, Japan

Correspondence and requests for materials should be addressed to H. K. (email: kaiju@appi.keio.ac.jp).

In this Supplementary Information section, we first present the voltage dependence of TMC curves at 60 and 40 kHz in sample B. Next, we show the voltage dependence of TMC and TMR in sample A. Finally, we provide the fitting parameters obtained in the calculation of frequency and bias voltage dependence of TMC for both samples A and B.

Voltage dependence of TMC curves at 60 and 40 kHz in sample B. Figure S1 shows the voltage dependence of TMC curves under a bias voltage of -450, 0, 100 and 500 mV at 60 Hz, and -500, -250, 50 and 200 mV at 40 kHz, respectively. At 60 Hz, the TMC ratio increases from 171% to 207% with increasing the positive bias voltage from 0 to 100 mV. Above 100 mV, the TMC decreases from 211% to 153% with increasing the voltage to 500 mV. In the negative bias region, the TMC decreases to 114% with decreasing the voltage to -450 mV. At 40 kHz, the TMC decreases from 6.88% to 5.72% with increasing

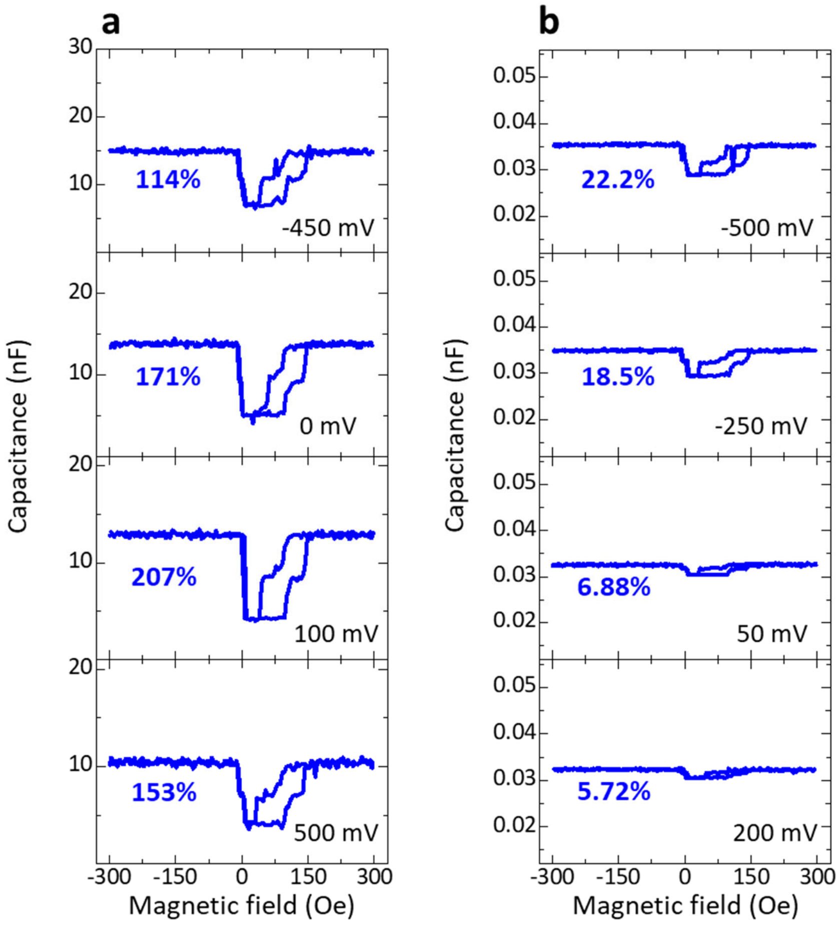

Figure S1 | Voltage dependence of TMC curves in sample B. (a) 60 Hz and (b) 40 kHz.

the positive voltage from 50 to 200 mV. As shown in Fig. 5(c) of the main text, above 200 mV, the TMC increases to 13.5% with increasing the voltage to 500 mV. In the negative bias region, the TMC increases up to 22% with decreasing the voltage to -500 mV. The experimental data are in good agreement with the calculation results.

Voltage dependence of TMC and TMR in sample A. Figure S2 shows the voltage dependence of TMC and TMR curves at 60, 140, and 40 kHz in sample A. At 60 Hz, the TMC ratio increases from 176% to 191% with increasing the positive bias voltage from 0 to 100 mV. Above 100 mV, the TMC decreases to 166% with increasing the voltage to 300 mV. In the negative bias region, the TMC decreases to 141% with decreasing the voltage to -150 mV. At 140 Hz, the TMC increases from 230% to 258% with increasing the positive voltage from 0 to 300 mV. In the negative bias region, the TMC decreases to 184% with decreasing the voltage to -200 mV. At 40 kHz, the TMC decreases from 9.64% to 6.93% with increasing the positive voltage from 0 to 100 mV. In the negative bias region, the TMC increases up to 15.1% with decreasing the voltage to -150 mV. The TMR decreases from 176% to 134% with increasing the voltage from 0 to 300 mV.

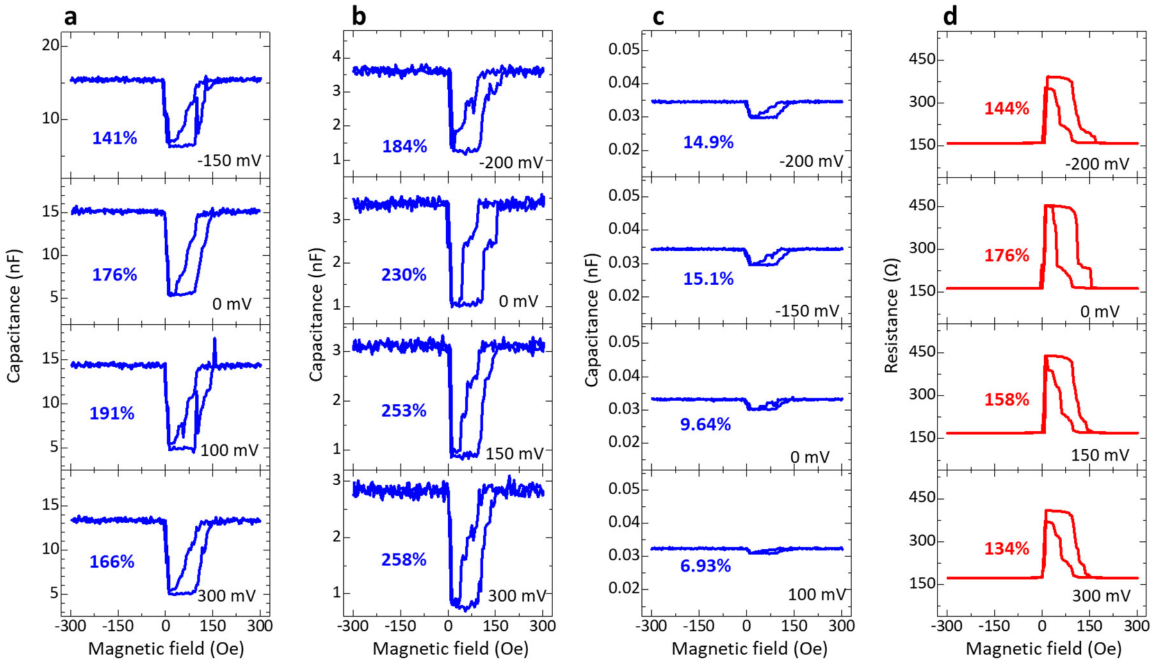

Figure S2 | Voltage dependence of TMC and TMR curves. TMC curves in an Fe/MAO/Fe MTJ (sample A) at (**a**) 60 Hz, (**b**) 140 Hz and (**c**) 40 kHz. (**d**) TMR curve in the same sample at 140 Hz.

Figure S3 shows the voltage dependence of TMC, TMR and the capacitance $C_{P(AP)}$ in the P(AP) configuration for sample A at 60 Hz, 140 Hz and 40 kHz, respectively. Experimental data provide excellent fits to the calculation results in the entire voltage region at each frequency.

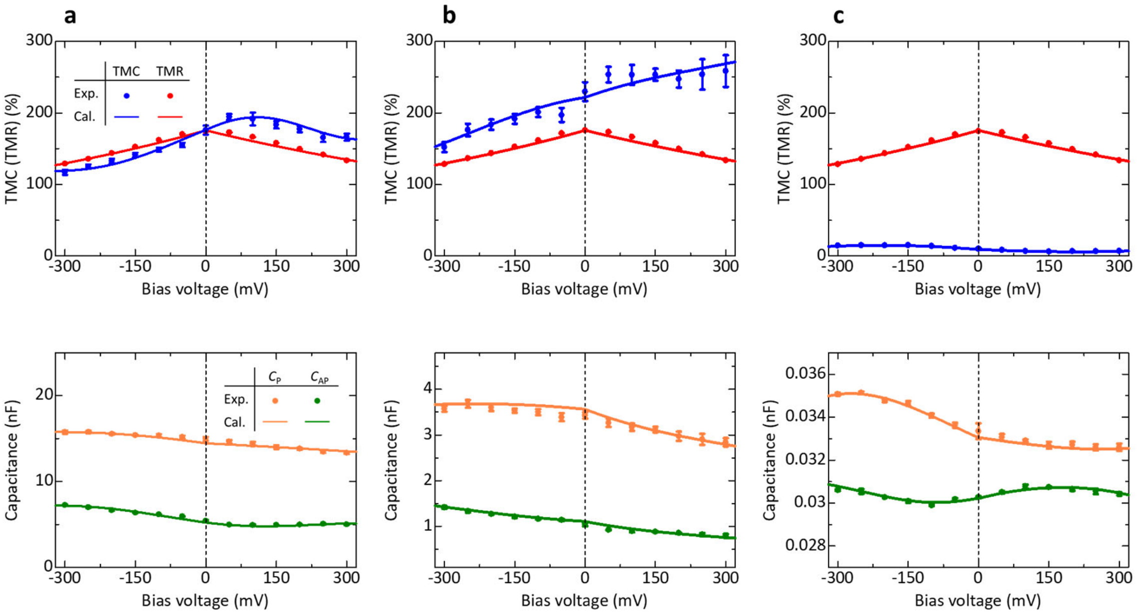

Figure S3 | Voltage dependence of TMC, TMR and $C_{P(AP)}$. TMC, TMR and the capacitance $C_{P(AP)}$ in the P(AP) configuration for an Fe/MAO/Fe MTJ (sample A) at (**a**) 60 Hz, (**b**) 140 Hz and (**c**) 40 kHz. The solid circles are experimental data and the solid lines represent the calculation results. The calculation is performed using SDD model and DF model combined with PBA and Zhang-sigmoid formula. There is an excellent agreement between the theory and experimental results.

Fitting parameters. The fitting parameters obtained in all the calculations for Fe/MAO/Fe MTJs (samples A and B) are shown in Tables S1−S3. $\phi_{0,P(AP)}$, its trend vs. frequency is different between the two samples although there is a slight difference in the nominal d_{MgAl}. The large difference is attributed to the significant difference in the bias dependence of TMC, shown in Fig. 5 and Fig. S3, respectively. As shown in Fig. S3, the jumped behavior in capacitance is not observed in sample A, whereas it is observed in sample B as shown in Fig. 5. The reason why such dramatic differences occur in each sample is not clear at the present stage, but they might be determined by the finished quality in each MTJ rather than the insulating thickness d_{MgAl}. Although these different behaviors cause a large difference in fitting results, the order of parameters is considered to be reasonable; $\phi_{0,P(AP)}$ = ~0.1−2 eV, $n_{P(AP)}$ = ~0.1−~10 cm^{-3}, and $V_{0,P(AP)}$ = ~0.3 V, which

are almost the same as previous results[1,2]. We also emphasize that experimental data are in good agreement with calculation results in the entire frequency and voltage regions shown in Fig. 3 and Fig. 5. The calculation results are obtained using Debye-Fröhlich (DF) model. According to DF-modelled calculation, we have achieved a large TMC of 155%[3] and 332%[1] in MgO-based MTJs, and 426% in MAO-based MTJs fabricated in this study. We have also discovered inverse TMC in Fe/Al-oxide/Fe_3O_4 MTJs[4] and sign inversion phenomenon of voltage-induced TMC in MgO-based MTJs[2]. From the good agreement between the experiments and calculations, the prediction reported in this study will allow us to observe a higher TMC in near future.

Table S1 Fitting parameters obtained in the calculation of frequency characteristics of TMC in samples A and B.

Sample	$C_{\infty,P(AP)}$ (nF)	$C_{0,P(AP)}$ (nF)	P	τ_P (mS)	$\beta_{P(AP)}$
A	0.0335 (0.0300)	47.25 (55.25)	0.594	4.0	0.9999 (0.9950)
B	0.0383 (0.0313)	42.25 (54.73)	0.627	3.9	0.9995 (0.9880)

Table S2 Fitting parameters obtained in the voltage dependence of TMC in sample A.

f (Hz)	Bias polarity	$K_{P(AP)}$ (V^{-1})	$\alpha_{P(AP)}$	$V_{0,P(AP)}$ (V)	$\phi_{0,P(AP)}$ (eV)	$n_{P(AP)}$ ($\times 10^{23} cm^{-3}$)
60	Positive	0.35 (5.0)	1.22 (9.4)	0.315 (0.30)	0.25 (0.9)	18.1 (4.2)
	Negative	1.35 (3.8)	1.22 (3.89)	0.315 (0.19)	0.2 (0.3)	18.1 (6.7)
140	Positive	1.3 (2.2)	3.3 (3.89)	0.36 (0.37)	1.1 (1.0)	3.1 (0.63)
	Negative	1.35 (1.4)	1.22 (3.89)	0.18 (0.153)	0.7 (1.1)	5.5 (3.8)
40k	Positive	19 (13)	5.56 (111.5)	0.27 (0)	1.5 (0.0447)	0.205 (0.0097)
	Negative	22 (34)	111 (13.9)	0 (0.09)	0.0587 (0.4)	0.0146 (0.064)

Table S3 Fitting parameters obtained in the voltage dependence of TMC in sample B.

f (Hz)	Bias polarity	$K_{P(AP)}$ (V^{-1})	$\alpha_{P(AP)}$	$V_{0,\ P(AP)}$ (V)	$\phi_{0,P(AP)}$ (eV)	$n_{P(AP)}$ ($\times 10^{23} cm^{-3}$)
60	Positive	1.3 (4.0)	3.3 (10.6)	0.090 (0.347)	1.3 (1.0)	15.6 (4.0)
	Negative	0.03 (0.6)	844 (0.56)	0.27 (0.36)	0.52 (0.07)	91.7 (3.3)
140	Positive	2.3 (25)	4.2 (1200)	0.180(0.31)	2 (1.8)	2.16(0.48)
	Negative	0.25 (0.70)	1.22 (956)	0.180 (0.153)	1.00 (0.140)	29.2 (2.05)
40k	Positive	21.5 (14.0)	6.22 (222)	0.270 (0.00)	1.52 (0.092)	0.165 (0.0193)
	Negative	15.0 (5.0)	22.3 (223)	0.001 (0.005)	0.10 (0.146)	0.025 (0.029)

References

1. Ogata, K., Nakayama, Y., Xiao, G. & Kaiju, H. Observation and theoretical calculations of voltage-induced large magnetocapacitance beyond 330% in MgO-based magnetic tunnel junctions. *Sci. Rep.* **11**, 13807 (2021).
2. Nakagawa, T., Ogata, K., Nakayama, Y., Xiao, G. & Kaiju. H. Sign inversion phenomenon of voltage-induces tunnel magnetocapacitance. *Appl. Phys. Lett.* **118**, 182403 (2021).
3. Kaiju, H. *et al*. Large magnetocapacitance effect in magnetic tunnel junctions based on Debye-Fröhlich model. *Appl. Phys. Lett.* **107**, 132405 (2015).
4. Kaiju, H. *et al*. Inverse tunnel magnetocapacitance in Fe/Al-oxide/Fe_3O_4. *Sci. Rep.* **7**, 2682 (2017).
